# Studies on the antidiarrhoeal activity of *Aegle marmelos *unripe fruit: Validating its traditional usage

**DOI:** 10.1186/1472-6882-9-47

**Published:** 2009-11-23

**Authors:** S Brijesh, Poonam Daswani, Pundarikakshudu Tetali, Noshir Antia, Tannaz Birdi

**Affiliations:** 1 The Foundation for Medical Research, 84A, R. G. Thadani Marg, Worli, Mumbai 400018, Maharashtra, India; 2 Naoroji Godrej Centre for Plant Research, Lawkin Ltd. Campus, Shindewadi, Shirwal, Satara 412801, Maharashtra, India; 3 The Foundation for Research in Community Health, 3-4, Trimiti-B Apartments, 85, Anand Park, Pune 411 007, Maharashtra, India

## Abstract

**Background:**

*Aegle marmelos *(L.) Correa has been widely used in indigenous systems of Indian medicine due to its various medicinal properties. However, despite its traditional usage as an anti-diarrhoeal there is limited information regarding its mode of action in infectious forms of diarrhoea. Hence, we evaluated the hot aqueous extract (decoction) of dried unripe fruit pulp of *A. marmelos *for its antimicrobial activity and effect on various aspects of pathogenicity of infectious diarrhoea.

**Methods:**

The decoction was assessed for its antibacterial, antigiardial and antirotaviral activities. The effect of the decoction on adherence of enteropathogenic *Escherichia coli *and invasion of enteroinvasive *E. coli *and *Shigella flexneri *to HEp-2 cells were assessed as a measure of its effect on colonization. The effect of the decoction on production of *E. coli *heat labile toxin (LT) and cholera toxin (CT) and their binding to ganglioside monosialic acid receptor (GM1) were assessed by GM1-enzyme linked immuno sorbent assay whereas its effect on production and action of *E. coli *heat stable toxin (ST) was assessed by suckling mouse assay.

**Results:**

The decoction showed cidal activity against *Giardia *and rotavirus whereas viability of none of the six bacterial strains tested was affected. It significantly reduced bacterial adherence to and invasion of HEp-2 cells. The extract also affected production of CT and binding of both LT and CT to GM1. However, it had no effect on ST.

**Conclusion:**

The decoction of the unripe fruit pulp of *A. marmelos*, despite having limited antimicrobial activity, affected the bacterial colonization to gut epithelium and production and action of certain enterotoxins. These observations suggest the varied possible modes of action of *A. marmelos *in infectious forms of diarrhoea thereby validating its mention in the ancient Indian texts and continued use by local communities for the treatment of diarrhoeal diseases.

## Background

India has a rich heritage of traditional knowledge and is home to several important time-honored systems of health care like Ayurveda, Siddha and Unani. It has been estimated that the proportion of medicinal plants in India (7,500 of the 17,000 higher plant species are medicinal plants) is higher than any country of the world with respect to the existing flora of that respective country [1,2].

*Aegle marmelos *(L.) Correa commonly known as *Bael*/*Bilva *belonging to the family Rutaceae has been widely used in indigenous systems of Indian medicine due to its various medicinal properties. Although this plant is native to northern India it is also widely found throughout the Indian peninsula and in Ceylon, Burma, Thailand and Indo-China. *A. marmelos *tree is held sacred by Hindus and offered in prayers to deities Lord Shiva and Parvati and thus the tree is also known by the name *Shivaduma *(the tree of Shiva) [3]. Hindus also believe that goddess Lakshmi resides in *Bael *leaves. It is therefore widely cultivated and commonly found in the vicinity of temples.

All parts of this tree, viz. root, leaf, trunk, fruit and seed are useful in several ailments. The root is an important ingredient of the '*Dasmula*' (ten roots) recipe [4]. The decoction of the root and root bark is useful in intermittent fever, hypo-chondriasis, melancholia, and palpitation of the heart [5]. The leaves and bark have been used in medicated enema. The leaves are also used in diabetes mellitus. The greatest medicinal value, however, has been attributed to its fruit [4] and the unripe fruit is said to be an excellent remedy for diarrhoea and is especially useful in chronic diarrhoeas [4-6]. The effectiveness of *A. marmelos *fruit in diarrhoea and dysentery has resulted in its entry into the British Pharmacopoeia [4]. Moreover, Chopra [4] has appropriately stated that "No drug has been longer and better known nor more appreciated by the inhabitants of India than the *Bael *fruit." Charaka has described this plant as a *Rasayana *[7].

Despite the traditional use of *A. marmelos *unripe fruit as an antidiarrhoeal, few studies have reported its antidiarrhoeal activity. According to Chopra [4], *A. marmelos *is effective in chronic cases of diarrhoea due to the presence of large quantities of mucilage, which act as a demulcent. Additionally, *A. marmelos *has been shown to be effective in experimental models of irritable bowel syndrome and physiological diarrhoea [8-10]. However, to the best of our knowledge, besides antiprotozoal studies [11], effect of *A. marmelos *unripe fruit in infectious diarrhoea has not been reported.

The pathogenesis of infectious diarrhoea has been widely studied. Enteric pathogens have evolved a remarkable array of virulence traits that enable them to colonize the intestinal tract. These organisms colonize and disrupt intestinal function to cause mal-absorption or diarrhoea by mechanisms that involve microbial adherence and localized effacement of the epithelium, production of toxin(s) and direct epithelial cell invasion [12]. Adherence which is a means of colonizing the appropriate ecological niche enables the organism to resist being swept away by mucosal secretions. Adherence also aids in subsequent proliferation and colonization of the gut and may be followed by toxin production or invasion [13]. The importance of using colonization and production and action of enterotoxins as specific parameters reflecting the pathogenesis has been earlier used by us as an approach towards understanding the varied mechanism(s) of action of antidiarrhoeal medicinal plants against infectious diarrhoea [14-17]. The studies highlighted the necessity of including multiple parameters for assessing effectiveness of medicinal plants against infectious forms of diarrhoea, especially in the absence of antimicrobial activity. Thus our studies deviate from a number of other studies that are restricted to intestinal motility and antibacterial activity as markers for antidiarrhoeal activity [18-29]. In this study, we evaluated the decoction of dried unripe fruit pulp of *A. marmelos *for its effect on various parameters of diarrhoeal pathogenicity, viz., adherence to and invasion of intestinal epithelium and production and action of enterotoxins to elucidate its mechanism(s) of action in infectious diarrhoea.

## Methods

### Plant material and preparation of decoction

A February collection of the unripe fruits of *A. marmelos *collected from Pangare village in the Parinche valley, about 53 km south east of Pune city in the state of Maharashtra, India, was used for the present study. February was chosen as the month of collection since the fruits achieve full size at this time but are still unripe. The plant material was authenticated by Dr. P. Tetali, Head, Research and Development, Naoroji Godrej Centre for Plant Research, Shirwal, Maharashtra, India. A voucher specimen has been deposited at the Botanical Survey of India (Western Circle), Pune, India, under herbarium number 124675. The fruits were cut into small pieces, shade dried and stored at 4°C.

A crude aqueous extract (decoction) was used for the study since it represents the nearest form to traditional preparations. The decoction was prepared as described in Ayurvedic text [30]. 1 g of the powdered dried fruit pulp was boiled in 16 ml double distilled water till the volume reduced to 4 ml. It was centrifuged and filtered through a 0.22 μm membrane before use. To replicate field conditions, each assay was performed with freshly prepared decoction.

The dry weight of the decoction thus obtained was 51.1 mg/ml ± 0.5 mg/ml and 20.4% ± 2.11% (w/w) with respect to the starting dried plant material. The decoction was diluted 1:100, 1:20 and 1:10 in appropriate media for each experiment and has been referred to as 1%, 5%, and 10% respectively throughout the text. The dry weight contents of these dilutions of the decoction were 0.51 mg/ml ± 0.005 mg/ml, 2.55 mg/ml ± 0.025 mg/ml and 5.11 mg/ml ± 0.05 mg/ml respectively.

### Media, reagents, plastic ware and instrumentation

The bacterial media and the Minimal Essential Medium (MEM) were purchased from HiMedia laboratory, Mumbai, India. Dulbecco's Modified Eagle's Medium (DMEM) and fetal calf serum (FCS) were procured from GibcoBRL, UK. The constituents of the Diamond's TYI-SS medium were procured from local Indian manufacturers, as were the antibiotics. Trypan blue, neutral red, ganglioside monosialic acid (GM1), anti-cholera toxin, ortho-phenylene diamine, bovine serum and bovine serum albumin were purchased from Sigma, USA. Peroxidase labeled swine anti-rabbit immunoglobulin was purchased from Dako, Denmark. All chemicals were from SD Fine Chemicals, Mumbai. Standard marmelosin was purchased from Natural Remedies, Bangalore, India. Gallic acid was kindly provided by Dr. KS Laddha, University Institute of Chemical Technology, Mumbai, India. Lactulose was a product of Intas Pharmaceuticals, Ahmedabad, India. The 24- and 96-well tissue culture plates and the 96-well ELISA plates were purchased from Nunclon, Denmark, the 55 mm diameter tissue culture plates were obtained from Tarsons, Kolkata, India, and the ELISA plate reader was purchased from Labsystems, Finland.

### Cell culture

The human laryngeal epithelial cell line, HEp-2, and the embryonic monkey kidney derived cell line, MA-104, were obtained from National Centre for Cell Sciences, Pune, India. The cell lines were maintained in DMEM and MEM respectively, supplemented with 10% FCS, at 37°C in a 5% CO_2 _atmosphere. The cells were maintained in logarithmic growth by passage every 3-4 days.

### Microorganisms used

Six bacteria, viz., enteropathogenic *Escherichia coli *(EPEC) strain B170, serotype 0111:NH, enterotoxigenic *E. coli *(ETEC) strains B831-2, serotype unknown (heat labile toxin producer) and strain TX1, serotype 078:H12 (heat stable toxin producer) (all strains obtained from Centre for Disease Control, Atlanta, USA), enteroinvasive *E. coli *(EIEC) strain E134, serotype 0136:H- (kindly provided by Dr. J. Nataro, Veterans Affairs Medical Centre, Maryland, USA), *Vibrio cholerae *C6709 El Tor Inaba, serotype 01 (kindly provided by Dr. S. Calderwood, Massachusetts General Hospital, Boston, USA) and *Shigella flexneri *M9OT, serotype 5 (kindly provided by Dr. P. Sansonetti, Institut Pasteur, France) were used for the present study. In addition, *Giardia lamblia *P1 trophozoites (kindly provided by Dr. P. Das, National Institute for Cholera and Enteric Diseases, Kolkatta, India) and simian rotavirus SA-11 (kindly provided by Dr. S. Kelkar, National Institute of Virology, Pune, India) were also included.

### Phytochemical analysis

Qualitative phytochemical analysis of the decoction was carried out for assaying presence of carbohydrates, glycosides, proteins, amino acids, phytosterols, saponins, flavonoids, alkaloids and tannins [31]. High performance thin layer chromatography (HPTLC) fingerprinting of the methanol soluble fraction of the decoction was carried out with the solvent system n-Hexane:Ethyl acetate:Acetic acid (40:60:0.5). Marmelosin was used as a phytochemical reference standard for fingerprinting.

### Antimicrobial activity

#### Antibacterial activity

The antibacterial activity was determined by a microtitre plate-based assay [32]. The bacterial strains were incubated in the absence (control) and presence of different dilutions of the decoction in nutrient broth and the optical density was measured after 24 h as a measure of growth. Three independent experiments were carried out. In each experiment, triplicate wells were set up for control as well as each dilution of the decoction.

#### Antigiardial activity

A 24 h culture of *G. lamblia *P1 trophozoites was incubated in the absence (control) and presence of different dilutions of the decoction in Diamond's TYI-SS medium. The number of viable trophozoites after 24 h was counted in a haemocytometer using trypan blue [33]. Three independent experiments were carried out. In each experiment, duplicate tubes were set up for control as well as each dilution of the decoction.

#### Antirotaviral activity

The entry and subsequent survival of rotavirus SA-11 in MA-104 cells was assayed by the neutral red uptake assay [34]. Briefly, MA-104 cells were grown in 96-well tissue culture plates for 72 h after which they were infected with rotavirus for 90 min in absence (control) and presence of different dilutions of the decoction. Subsequently, the extracellular virus and the decoction were washed off and the culture was further incubated for 72 h. Thereafter, the cells were incubated with 0.03% neutral red dye for 30 min. The intracellular dye was released with 1:1 solution of 100 mM acetic acid and ethanol and the intensity measured at 540 nm (reference 630 nm) in an ELISA plate reader. Three independent experiments were carried out. In each experiment, triplicate wells were set up for control as well as each dilution of the decoction.

### Effect on bacterial colonization

#### Effect on adherence

The effect on the adherence of *E. coli *strain B170 to epithelial cells was assayed by the method described by Cravioto *et al*. [35]. Briefly, a 48 h culture of HEp-2 cells on glass coverslips was infected with a log phase culture of the bacterium (5 × 10^7^/ml) and incubated for 3 h. Non-adherent bacteria were washed off, the coverslips were fixed in 10% formaldehyde and stained with toluidine blue stain (0.1% w/v). HEp-2 cells having typical EPEC micro-colonies [36] were counted under light microscope. Three independent experiments were carried out. In each experiment, duplicate cover-slips were set up for control as well as each dilution of the decoction.

#### Effect on invasion

The effect on invasion of *E. coli *E134 and *S. flexneri *into epithelial cells was studied by the method described by Vesikari *et al*. [37]. Briefly, a 48 h culture of HEp-2 cells grown in a 24-well tissue culture plate was infected with log phase culture of the bacteria (10^8^/ml) and incubated for 2 h. The culture was further incubated with gentamycin (100 μg/ml) for 3 h. The epithelial cells were then lysed by cold shock with chilled distilled water and the released bacteria were counted by plating on nutrient agar. Three independent experiments were carried out. In each experiment, duplicate wells were set up for control as well as each dilution of the decoction.

Two different protocols were performed for both the adherence and the invasion assays to understand whether the bacterial adherence and invasion respectively were affected by the effect of the decoction on the epithelial cells or through competitive inhibition. The HEp-2 cells were incubated in absence (control) and presence of different dilutions of the decoction either for 18-20 h prior to infection (pre-incubation) or simultaneously with infection (competitive inhibition) respectively.

### Effect on bacterial enterotoxins

#### Effect on *E. coli *heat labile toxin (LT) and cholera toxin (CT)

LT, which is localized in the bacterial cell membrane, was obtained by lysing *E. coli *B831-2 with polymyxin B sulphate (1 mg/ml) whereas CT, which is released extracellularly, was obtained as a culture supernatant of *V. cholerae*. LT and CT were assayed by the GM1-enzyme linked immunosorbent assay (GM1-ELISA) [38]. Briefly, the toxins were added to ELISA plates pre-coated with 1.5 μmol/ml of GM1. Anti-cholera toxin and peroxidase labeled swine anti-rabbit immunoglobulin used at dilutions of 1:300 and 1:200 were used as primary and secondary antibodies respectively. Ortho-phenylene diamine (6 mg) with hydrogen peroxide (4 μl) in 10 ml citrate buffer (pH 5.5) was used as the substrate. The intensity of the color thus developed was read at 492 nm in an ELISA plate reader.

To study the effect on production of the toxins, the respective bacteria were grown in Casein Hydrolysate Yeast Extract (CAYE) in absence (control) and presence of different dilutions of the decoction and the toxins produced were assayed by GM1-ELISA. To study the effect on the binding of the toxins to GM1, the toxins obtained by growing the respective bacteria in CAYE were added to the assay system in absence (control) and presence of the decoction. Three independent experiments were carried out. In each experiment, triplicate wells were set up for control as well as each dilution of the decoction.

#### Effect on *E. coli *heat stable toxin (ST)

ST was assayed by the method originally described by Gianella [39]. Briefly, ST, which is released extracellularly, was obtained as the culture supernatant of *E. coli *TX1. The toxin was inoculated intra-gastrically in 2-3 days old Swiss White suckling mice. Following an incubation of 3 h at room temperature, the pups were sacrificed and the ratio of gut weight to that of the remaining carcass weight was calculated. Ratio of ≥ 0.083 was considered as positive.

To study the effect on production of ST, the bacterium was grown in CAYE in absence (control) and presence of different dilutions of the decoction and the toxin produced was assayed. To study the effect on the action of ST, the toxin obtained by growing the bacterium in CAYE was intra-gastrically injected in absence (control) and presence of different dilutions of the decoction. CT was used as a negative control. Three independent experiments were carried out. In each experiment, three animals were inoculated for control as well as each dilution of the decoction.

The Institutional Ethics Committee and the Committee for the Purpose of Control and Supervision of Experiments on Animals (CPCSEA) cleared the use of animals in the study. The Foundation for Medical Research (FMR) is registered with CPCSEA (registration No. 424/01/a/CPCSEA, June 20th, 2001).

### Statistical analysis and presentation of data

The results for each assay have been expressed as the mean ± standard error of the percentage values from three independent experiments. The percentage in each experiment was calculated using the formula {(C or T)/C} × 100, where C is the mean value of the duplicate/triplicate readings of the control group and T is mean value of the duplicate/triplicate readings of the test (dilutions of the decoction) groups. Hence, the value of control is 100% and the values of the test groups have been represented as percentages relative to control.

Data were analyzed by analysis of variance (ANOVA) and Dunnett's post-test. Statistical analyses were performed using the software Prism 4.0 (GraphPad, Inc.). *P *≤ 0.05 was considered to be statistically significant.

## Results

### Phytochemistry

**Figure 1:**
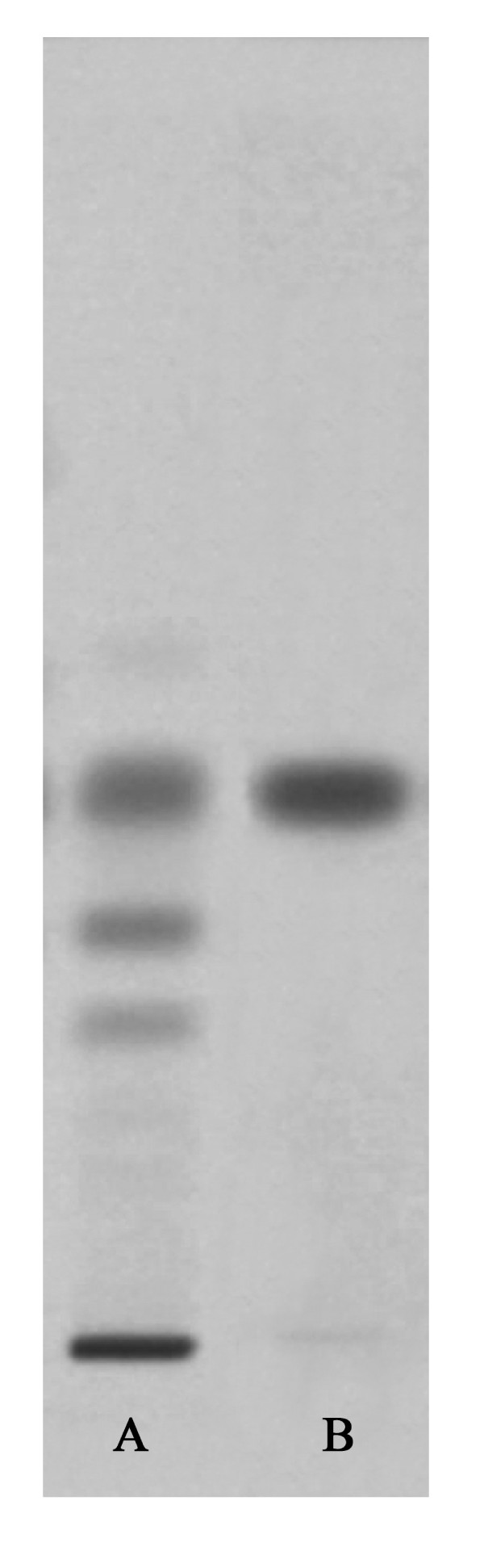
**HPTLC of the decoction of *A. marmelos***. (A) Methanol fraction of the decoction. (B) Marmelosin.

The decoction contained carbohydrates, glycosides, amino acids, proteins, tannins, flavanoids, and phytosterols. The results were similar to earlier reports [40,41]. The chromatogram of the HPTLC fingerprinting analysis of the methanol soluble fraction of the decoction scanned at 254 nm has been presented in Fig 1.

### Antimicrobial activity

**Figure 2:**
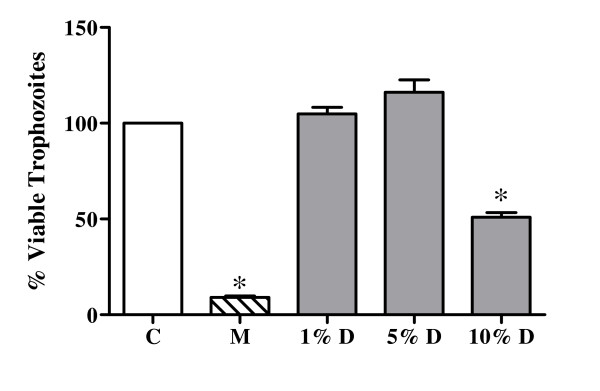
**Antigiardial activity of the decoction of *A. marmelos***. C: Control, trophozoites in medium alone; M: trophozoites incubated in medium with metronidazole (10 μg/ml); D: trophozoites incubated in medium with decoction. Values represent mean ± standard error (n = 3) of percentage viable trophozoites relative to control (100%); * *P *< 0.05.

In comparison to ofloxacin (1 μg/ml), which completely inhibited all the six bacterial strains tested, the decoction did not inhibit the growth of any of the bacteria (data not shown). The decoction, however, affected growth of *G. lamblia*. The number of viable trophozoites was significantly lower (approximately 50%) at 10% dilution of the decoction (Fig 2), as observed by trypan blue staining. The surviving trophozoites failed to multiply when provided with fresh medium indicating that *A. marmelos *was cidal for giardia. However, the decrease though statistically significant was less than that observed with metronidazole (10 μg/ml), which resulted in almost 90% killing of the trophozoites.

**Figure 3:**
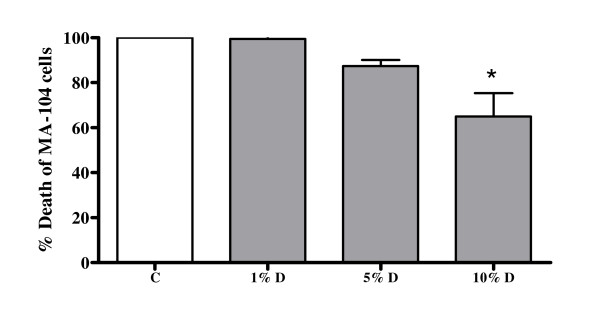
**Antirotaviral activity of the decoction of *A. marmelos***. C: Control, rotavirus infected MA-104 cells in medium alone; D: Rotavirus infected MA-104 cells in medium with decoction. Values represent mean ± standard error (n = 3) of percentage viable MA-104 cells relative to control (100%); * *P *< 0.05.

Rotavirus, following entry, lyses MA-104 cells and hence the number of viable cells remaining at the end of the assay is an indirect measure of antirotaviral activity of the decoction. It was observed that compared to the control, cell death was decreased following infection with the virus in the presence of 10% dilution of the decoction (Fig 3) indicating that the decoction was inhibitory to the virus at this dilution.

### Effect on bacterial colonization

**Figure 4:**
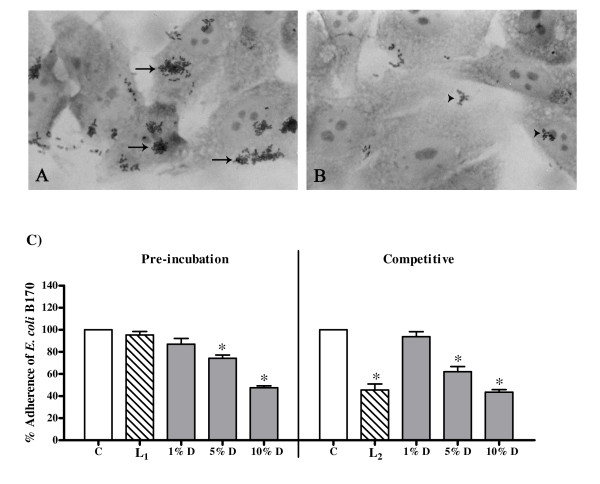
**Effect of the decoction of *A. marmelos *on bacterial adherence to HEp-2 cells**. (A) *E. coli *B170 microcolonies (arrows) on HEp-2 cells in medium alone. (B) *E. coli *B170 microcolonies (arrow heads) on HEp-2 cells when incubated in medium with 10% dilution of the decoction. (C) Adherence of *E. coli *B170 to the HEp-2 cells in the pre-incubation (HEp-2 cells incubated with the decoction prior to infection) and the competitive (HEp-2 cells incubated with the decoction simultaneously with the infection) protocols. C: Control, adherence to HEp-2 cells in medium alone; L_1_: Adherence to HEp-2 cells when pre-incubated in medium with 2.5 mg/ml lactulose; L_2_: Adherence to HEp-2 cells in medium with 15 mg/ml lactulose in the competitive protocol; D: Adherence to HEp-2 cells in medium with decoction. Values represent mean ± standard error (n = 3) of percentage adherence relative to control (100%); * *P *< 0.05.

Fig 4A shows characteristic micro-colony formation typical of EPEC whereas Fig 4B shows a representative image of the effect on the micro-colony formation on HEp-2 cells in presence of 10% dilution of the decoction. As can be seen in Fig 4B, the decoction was not cytotoxic to HEp-2 cells. The adherence of *E. coli *B170 to HEp-2 cells was significantly reduced by the decoction in both the protocols (Fig 4C).

The effect of the decoction on adherence of *E. coli *B170 to HEp-2 cells was compared with that of lactulose, a prebiotic oligosaccharide, known to inhibit adherence of EPEC to tissue culture cells [42]. As compared to the control the adherence of *E. coli *B170 to the HEp-2 cells in the presence of 10% dilution of the decoction in the competitive protocol was 43.42% ± 2.51% whereas it was 45.44% ± 5.44% in the presence of lactulose (15 mg/ml). In the pre-incubation protocol, lactulose was toxic to HEp-2 cells even at 5 mg/ml when incubated overnight with the cells. Below this concentration lactulose had no effect on the adherence. The decoction, on the other hand, showed 47.49% ± 1.82% adherence of *E. coli *B170 to HEp-2 cells at 10% dilution in the pre-incubation protocol.

**Figure 5:**
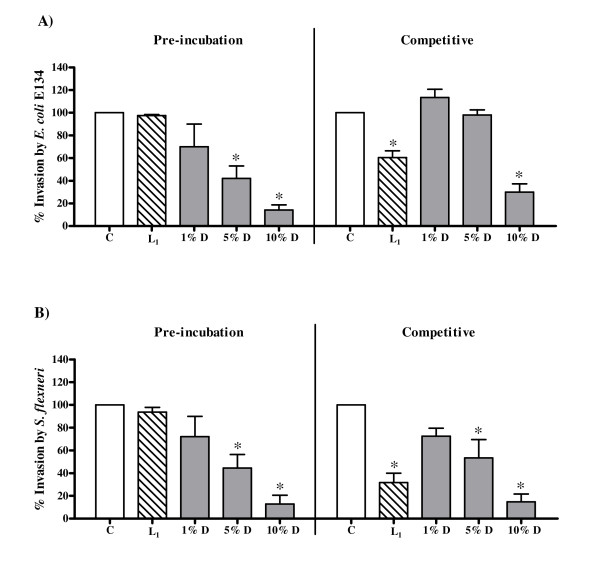
**Effect of the decoction of *A. marmelos *on bacterial invasion to HEp-2 cells**. (A) Invasion of *E. coli *E134 to HEp-2 cells in the pre-incubation (HEp-2 cells incubated with the decoction prior to infection) and the competitive (HEp-2 cells incubated with the decoction simultaneously with the infection) protocols. (B) Invasion of *S. flexneri *to HEp-2 cells in the pre-incubation (HEp-2 cells incubated with the decoction prior to infection) and the competitive (HEp-2 cells incubated with the decoction simultaneously with the infection) protocols. C: Control, invasion to HEp-2 cells in medium alone; L_1_: Invasion to HEp-2 cells in medium with 2.5 mg/ml lactulose; D: Invasion to HEp-2 cells in medium with decoction. Values represent mean ± standard error (n = 3) of percentage invasion relative to respective control (100%); * *P *< 0.05.

The decoction also significantly reduced the invasion of both *E. coli *E134 and *S. flexneri *in both protocols (Fig 5A and Fig 5B respectively). The effect of the decoction on invasion of *S. flexneri *was compared with that of lactulose, as it has been used for the treatment of shigellosis and inflammatory bowel disease [43]. Since the mechanism of invasion of both EIEC and *S. flexneri *is almost identical [44], the effect of the decoction on invasion of *E. coli *E134 was also compared with that of lactulose. The decoction showed maximum decrease in invasion at 10% dilution with 28.87% ± 7.37% invasion of *E. coli *E134 and 14.78% ± 6.84% invasion of *S. flexneri *in the competitive protocol. In comparison, lactulose (2.5 mg/ml) showed 60.38% ± 5.94% and 31.68% ± 8.29% invasion for *E. coli *E134 and *S. flexneri *respectively. In the pre-incubation protocol, as seen in the adherence assay, lactulose was found to be toxic to HEp-2 cells even at 5 mg/ml when incubated overnight with the cells. Below this concentration lactulose had no effect on the invasion of either strain to the HEp-2 cells. The decoction, on the other hand, showed 14.13% ± 4.65% and 12.93% ± 7.68% invasion of *E. coli *E134 and *S. flexneri *respectively to HEp-2 cells at 10% dilution in the pre-incubation protocol.

### Effect on bacterial enterotoxins

**Figure 6:**
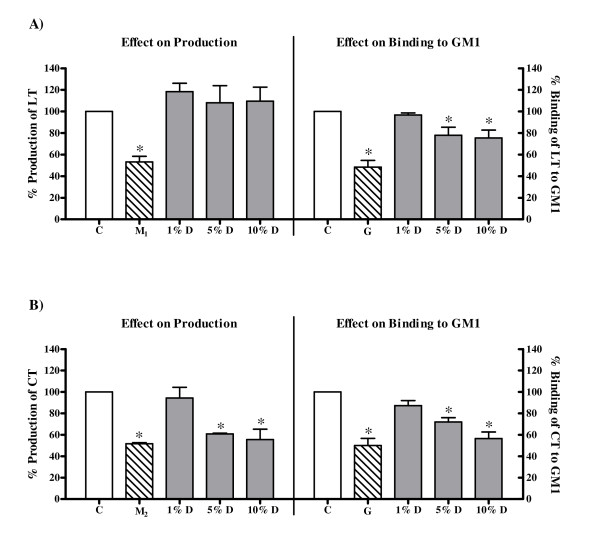
**Effect of the decoction of *A. marmelos *on bacterial enterotoxins**. (A) Production of *E. coli *heat labile toxin (LT) and its binding to GM1. (B) Production of cholera toxin (CT) and its binding to GM1. C: Control, toxin in medium alone; M_1_: LT in medium with 5 mM 2-mercaptoethanol; M_2_: CT in medium with 1 mM 2-mercaptoethanol; G: Toxin in medium with 50 mM gallic acid; D: Toxin in presence of decoction. Values represent mean ± standard error (n = 3) of percentage production/binding relative to respective control (100%); * *P *< 0.05.

On incubation of *V. cholerae *with the decoction, the production of CT was inhibited (Fig 6B). The decoction showed maximum inhibition of production of CT at 10% dilution with the percent production being 55.56% ± 9.72% compared to control. However, production of LT by *E. coli *B831-2 was not affected (Fig 6A). The effect of the decoction on production of these toxins was compared with that of 2-mercaptoethanol since thiols such as 2-mercaptoethanol, L-cysteine monohydrochloride and sodium thioglycolate have been reported to inhibit production of LT, CT and ST [45,46]. The production of LT and CT in presence of 2-mercaptoethanol (5 mM and 1 mM respectively) was 53.27% ± 5.30% and 51.63% ± 3.40% respectively. The bacterial growth was not affected at these concentrations of 2-mercaptoethanol (data not shown).

The binding of both LT (Fig 6A) and CT (Fig 6B) to GM1, on the other hand, was affected by the decoction. The decoction showed maximum inhibition at 10% dilution with the binding of these toxins to GM1 being 75.42% ± 7.34 and 56.58% ± 5.99% respectively compared to control. The effect of the decoction on binding of these toxins to GM1 was compared with that of gallic acid, a polyphenol, as it is reported to block the binding of LT to GM1 [47]. As LT and CT are antigenically closely related [48], the effect of the decoction on the binding of CT was also compared to that of gallic acid. The binding of LT and CT to GM1 in presence of gallic acid (50 mM) was 48.36% ± 6.35% and 50.04% ± 6.56% respectively.

The production and action of ST was not affected by the decoction at any of the dilutions tested (data not shown).

## Discussion

Diarrhoeal diseases are amongst the most common infectious diseases worldwide resulting in 3.2% of all deaths killing about 1.8 million people globally each year [49]. Annually, diarrhoeal diseases kill over 1.5 million children globally [50]. Even though economic development and progress in health care delivery are expected to catalyze substantial improvements in infectious disease related morbidity and mortality by the year 2020, it is predicted that diarrhoea will remain a leading health problem [51]. It affects mostly children in developing countries and can lead to dehydration and death and in survivors to impaired growth and malnutrition [52]. In adults, while the impact is less severe, it nevertheless can lead to nutritional deficiencies especially in the case of persistent diarrhoea [53].

*A. marmelos *has been used for centuries in India not only for its dietary purposes but also for its various medicinal properties [4-6]. The fruit is widely consumed as '*serbet*' (liquid fruit concentrate) and '*murbha*' (jam) and the unripe fruit is highly recommended for diarrhoea and is especially for chronic diarrhoea [3-6,54]. Hence, it is generally considered safe and few studies have been carried out with respect to its toxicity. Nevertheless, aqueous extract of *A. marmelos *fruit has been reported to be non mutagenic to *Salmonella typhimurium *strain TA 100 in the Ames assay [55]. In addition, acute toxicity studies have reported that a hydroalcoholic extract of *A. marmelos *fruit is non-toxic up to a dose of 6 g/kg body weight in mice [56]. Pharmacological studies on animal models involving repeated doses of *A. marmelos *fruit extract over a period of up to 30 days have not reported any adverse effect up to a maximum dose of 250 mg/kg body weight [56-59]. The decoction of *A. marmelos *showed no cytotoxic activity on HEp-2 cells in the present study even at the highest concentration tested (Fig. 4B).

Though a few studies have been carried out on the antidiarrhoeal activity of *A. marmelos *[8-10], no reports are available pertaining to its activity in infectious diarrhoea. The present work with the crude aqueous extract of dried unripe fruit pulp of *A. marmelos *provides an insight into its possible mechanism of action in infectious diarrhoea and validates its traditional use as an antidiarrhoeal. The study has intentionally been undertaken using a crude aqueous extract as it is our belief that the different biological activities assayed herein may not be due to a single constituent. This has also been highlighted by Mavar-Manga *et al*. [60] who have stated that crude extracts contain several compounds acting on different mechanisms. In addition, interplay of the constituents in the crude extract may result in better activity due to synergism or lead to decrease in toxicity and it is possible that pure compound(s) may not necessarily behave in the same manner as the natural extract [61,62].

The decoction of *A. marmelos *exhibited antigiardial and antirotaviral activity whereas it did not show any antibacterial activity. The results show that despite not being bactericidal, the antidiarrhoeal effect of this plant is possibly due to its ability to affect other bacterial virulence parameters.

*A. marmelos *prevented the colonization by *E. coli *B170, *E. coli *E134 and *S. flexneri*. The reduction in colonization is probably due to its effect on the metabolism of HEp-2 cells and/or modification of cell receptors to prevent adherence or bacterial entry as seen on the pre-incubation of HEp-2 with the decoction. The decoction exhibited greater inhibition of invasion of *E. coli *E134 and *S. flexneri *as compared to adherence of *E. coli *B170 in both protocols. This indicates that the decrease in invasion may not merely be due to the inhibition of initial attachment of the bacteria to the epithelial cells by the plant decoction but also could be due to its effect on the engulfment process of the bacteria at a post adherence stage. Thus the results of both adherence and invasive assays, representative of the colonization of the pathogens to the intestinal epithelium, indicate that *A. marmelos *does not permit the pathogens to establish themselves. It may be noted that since the adherence of the pathogen to the gut epithelium is the foremost stage of the disease process, inhibition of adherence could be a very important aspect in the antidiarrhoeal activity of the plant.

The decoction also reduced the binding of both LT and CT to the GM1 thereby inhibiting their action. LT and CT are known to be antigenically similar [48]. Hence, the effect of the decoction on their binding suggest that it may contain some compound(s), which either bind to the common antigenic moiety of these toxins or may directly block the GM1 on the cell membrane thereby inhibiting their binding to the receptor. In addition, though the decoction had no effect on production of LT it inhibited the production of CT. Since the decoction had no cidal activity against *V. cholerae*, suppression of CT production suggests that the decoction affected the bacterial metabolism.

Literature shows presence of mucilage, pectin, coumarins such as marmelosin and marmelide, and tannins in *A. marmelos *fruits [11,54,63,64]. In the current study, the qualitative phytochemical analysis of the decoction showed presence of carbohydrates, glycosides, amino acids, proteins, tannins, flavanoids, phytosterols and the HPTLC analysis showed the presence of marmelosin. Tannins and flavonoids in general have been reported to have antidiarrhoeal activity through inhibition of intestinal motility, antimicrobial action and antisecretory effects [20,23,24,26-28,65]. However, none of the isolated chemical constituents from the plant have been specifically studied for their antidiarrhoeal activity including effect on colonization and production and action of enterotoxins.

## Conclusion

The present study validates the use of unripe fruit of *A. marmelos *as an anti-diarrhoeal agent in traditional medicine. The results obtained in the study suggest that the decoction of *A. marmelos *can control several forms of infectious diarrhoeal diseases caused by EPEC, EIEC, LT producing ETEC, *V. cholerae, S. flexneri *and to some extent it can also control giardiasis and rotaviral infections. However, it may not be effective against diarrhoea caused by ST producing ETEC.

The study emphasizes that the bioassays used in the present study which represent intestinal pathology can be employed as possible novel targets for studying antidiarrhoeal activity of medicinal plants, especially in absence of antimicrobial activity. It, therefore, provides a new basis for the development of potent antidiarrhoeal therapy from medicinal plants. In addition, the study also highlights the importance of using relevant and where necessary multiple bioassays covering the entire spectrum of activities that can provide a more reliable evaluation of the biological efficacy of medicinal plants.

## Competing interests

The authors declare that they have no competing interests.

## Authors' contributions

BS and PD carried out the laboratory studies, helped in analysis of data and preparation of manuscript. PT collected the plant material, authenticated it and obtained a voucher specimen number. NA and TB were responsible for the study. All the authors except Late Dr. Noshir Antia have read and approved the final version of the manuscript.

## Pre-publication history

The pre-publication history for this paper can be accessed here:

http://www.biomedcentral.com/1472-6882/9/47/prepub
